# Fatal left cardiac failure caused by external compression of left internal mammary artery graft in an accident: a case report

**DOI:** 10.4076/1757-1626-2-8067

**Published:** 2009-08-25

**Authors:** Bulent Uslu, Mie Nielsen, Henrik Schmidt, Morten Hansen, Michael D Nielsen

**Affiliations:** 1 Department of Anaesthesia and Intensive Care Unit, Odense University HospitalSdr. Boulevard 29, 5000 Odense CDenmark; 2 Department of Radiology, Odense University HospitalSdr. Boulevard 29, 5000 Odense CDenmark

## Abstract

We report for the first time a case of a 54 years old man with a fatal motorcycle accident due to an external bleeding compression of left internal mammary artery graft to the left anterior descending artery. The possibility of cardiac failure in trauma caused by external compression or traumatic injury of the graft should be considered in people who previously underwent coronary artery bypass graft surgery.

## Introduction

External compression of the left internal mammary artery (LIMA) or traumatic injury to this graft should be considered as a possible cause of myocardial infarction in people, who previously underwent coronary artery bypass graft (CABG) surgery. The anatomical location of LIMA along inner side of the anterior chest wall, close to sternum makes it susceptible to traumatic injury and repeated chest surgery. LIMA is usually grafted to the left anterior descending artery (LAD). Interruption of blood supply from this artery might cause ischemic failure of the left myocardium after CABG. This case is the first of which we are aware in which a motorcyclist dies after an accident, because of external bleeding compression of a LIMA-graft. Left cardiac failure was probably the cause of his death and was later confirmed by autopsy.

## Case presentation

**Figure 1.**
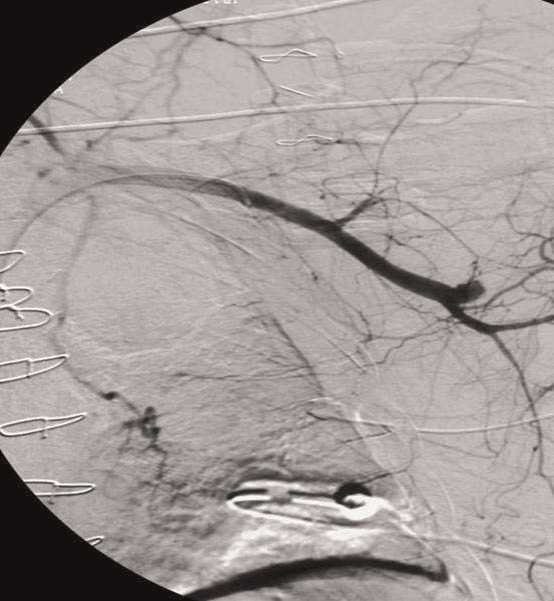
Angiography shows open LIMA-graft to LAD.

A 54 years old Danish motorcyclist collided with a car at full speed. He was transferred to the trauma centre after initial stabilisation by advanced trauma life support principles including at the scene intubation. Despite intensive transfusions, he was haemodynamically unstable upon arrival. The injuries included a traumatic amputation of the left forearm and lower leg, an unstable fracture of the pelvis, arterial bleeding from the upper left arm, and had an enormous haematoma around the left clavicle. The pelvic fracture was fixed with a c-clamp and his left leg was surgically amputated at knee level, while the left arm was amputated at the middle of the upper arm. The arterial bleeding originated from the left brachial artery and from one of the side branches of the subclavian artery, and these were coiled at the Department of Radiology. In the same session the angiography visualised the open left LIMA graft to the LAD (Figure 1). After initial stabilization further bleeding elsewhere was excluded by computer tomography (CT) contrast scan. This trauma CT-scan confirmed also that the haematoma above the left clavicle, haemothorax in the apical side of the left lung and a 3 cm dislocation of the left sterno-clavicular articulation. There were several fractures of the upper ribs anteriorly. The initial treatment of haemothorax with two pleural tubes drainage failed to improve the condition, even though the correct location of tubes was confirmed by X-ray. After initial treatment, including surgical interventions, the patient was transferred to the ICU with no immediate need for further intensive volume infusion.

Gradually the condition deteriorated during the following hours and cardiovascular instability developed. A 3 lead electrocardiogram (ECG) on screen showed neither ischemia nor arrhythmia. ECG with 12-lead was not taken at the arrival to the hospital. Volume therapy was insufficient and Dopamine and Epinephrine infusions were instituted. Other sources of bleeding were excluded by re-evaluation by the trauma team. Treatment with NovoSeven (Recombinant Coagulation Factor VIIa) did not improve patient stability. The next day, about 20 hours after the accident, the patient developed critically low blood pressure despite volume and inotropic treatment. A transthoracic echocardiography (TTE) at this time showed a hypokinetic anterior left side of the myocardium, which was supplied from the LIMA-LAD graft. Left ventricle ejection fraction (LVEF) was 30 %. There was neither pericardium exudation nor valvulopathia of aortic and mitral valves. Repeated chest X-ray showed that the subcutaneous haematoma around the left clavicle had increased in size.

The patient suffered cardiac arrest with asystole about 24 hours after the accident and resuscitation was unsuccessful. The autopsy showed new infarction of the left anterior myocardium supplied by the LIMA graft, which were without direct traumatic injury. The apical part of left lung was crushed with a large amount of coagulated blood at this area. Fractures of left ribs (1-4 ribs) were found as well as severe dislocation of the left sterno-clavicular joint.

## Discussion

LIMA has been utilized as a graft to LAD for many years because of the superiority to venous grafts. But graft failure is possible during the bypass surgery, when it is mobilized, prepared and connected to the LAD. It is well known that kinks of a tortuous LIMA graft can cause myocardial ischemia [1,2]. The flexibility of the intra coronary stent might improve these kinks in the short and long term, but surgical correction is an important option [3].

The long term patency of LIMA makes it also susceptible to graft failure due to injury or surgical procedures in chest. Chou et al. reported a case with acute myocardial infarction (AMI) caused by external compression of LIMA- graft during implantation of pacemaker leads in the subclavian vein, as the leads passed LIMA graft [4]. Others have reported AMI and cardiogenic shock despite of systemic inotropic, caused by compression of LIMA graft by mediastinal draining tube [5,6]. There was an immediate resolution of AMI, when the draining tube was removed. Accidental puncture of LIMA-graft has also been reported in cases, when insertion of central venous catheter has occurred to subclavian vein left side [7]. Repeated bypass operation and valve surgery might result in graft injury and entrapment of the LIMA graft caused by sternal wires has been reported [8].

Coronary subclavian steal syndrome (CSSS) is another cause of AMI or angina pectoris as reported by Crawly et al. [9]. His team found out that a left arteriovenous hemodialysis fistula will draw the blood flow away from the LIMA, because of higher resistance in the stenotic LAD and further drawing of blood during the dialysis might cause angina pectoris. CSSS is also plausible in stenosis or coiling of left proximal subclavian artery because of reversal blood flow from LIMA to the vertebral artery [10].

## Conclusion

In this case left cardiac failure was the cause of death despite massive Adrenaline infusion and volume therapy confirmed by the fresh infarctions in left ventricle in the autopsy. But it is uncertain, whether the infarction was caused by the external compression of the size increasing haematoma to the proximally part of LIMA graft, or by the compression of the two chest draining tubes in the upper left side. These tubes were placed in the apical part of the pleura to drain the haemothorax, but without any success.

The CSSS might also be a plausible cause in our case. The coiling of the side branch of the subclavian artery and the detecting thrombosis in the distal part of subclavian artery might cause retrograde thrombosis of blood in the LIMA graft or CSSS, as the blood flow will be away from the LIMA graft. Another possible cause of LIMA occlusion is lesions of intima layer inside these vessels.

It is difficult to assume only one reason of the fatal result of this case. But the visualizing of the LIMA graft to the LAD at the angiography initially and the gradual deterioration of cardiac failure as the haematoma proximally to the LIMA graft increased in size, suggest that external compression of LIMA was the cause of AMI and cardiac failure. Missing of the 12 lead ECG and the cardiac monitoring with only 3 lead ECG in this case might have made it difficult to recognize ischemia. In conclusion, external compression of the LIMA or traumatic injury to this graft should be considered as a possible cause of myocardial infarction in chest trauma. To our knowledge, this case about traumatic effect of LIMA graft after an accident has not yet been described elsewhere.

## Abbreviations

AMI: acute myocardial infarction
CABG: coronary artery bypass graft
CSSS: coronary subclavian steal syndrome
CT: computer tomography
ECG: electrocardiogram
ICU: intensive care unit
LAD: left anterior descending artery
LIMA: left internal mammary artery
LVEF: left ventricle ejection fraction
TTE: transthoracic echocardiography
